# Development of a TaqMan-Based Duplex Real-Time Quantitative RT-PCR for Detection and Differentiation of Muscovy Duck Reovirus and Novel Duck Reovirus

**DOI:** 10.3390/pathogens14121231

**Published:** 2025-12-02

**Authors:** Li Liu, Jinping Fu, Mengzhou Lin, Anping Wang, Shuang Wu, Chuanmin Liu

**Affiliations:** 1 School of Veterinary Medicine and Public Health, Jiangsu Agri-Animal Husbandry Vocational College, Taizhou 225300, China; vetlily@163.com (L.L.); linmengzhouabc@163.com (M.L.); wap4017@163.com (A.W.); 2 Institute of Veterinary Medicine, Jiangsu Academy of Agricultural Sciences, Nanjing 210014, China; 3 Key Laboratory of Veterinary Biological Engineering and Technology, Ministry of Agriculture and Rural Affairs, Nanjing 210014, China; 4 College of Animal Science, Tibet Agriculture and Animal Husbandry University, Nyingchi 860000, China; 5 College of Veterinary Medicine, Yangzhou University, Yangzhou 225009, China; 6 College of Animal Science and Food Engineering, Jinling Institute of Technology, Nanjing 210038, China; 7 School of Medicine, Linyi University, Linyi 276000, China; 8 School of Life Sciences, Jiangsu University, Zhenjiang 212013, China; 9 College of Veterinary Medicine, Nanjing Agricultural University, Nanjing 210014, China

**Keywords:** muscovy duck reovirus (MDRV), novel duck reovirus (NDRV), TaqMan probe, real-time PCR

## Abstract

Muscovy duck reovirus (MDRV) and novel duck reovirus (NDRV) are major pathogens in duck breeding, leading to substantial economic losses in the waterfowl industry. This study aimed to develop a precise detection and differentiation method for both viruses simultaneously. Specific primers and probes targeting the S3 gene were designed, and a duplex TaqMan-based real-time RT-PCR assay was established following optimization of reaction conditions. The assay demonstrated high amplification efficiency (100.1–106.7%), strong linear correlation (R^2^ > 0.999), and low limits of detection (13–25 copies/µL). Intra- and inter-assay coefficients of variation were below 1.5%, confirming excellent repeatability and stability. Applied to 122 clinical duck tissue samples, the assay detected MDRV in 29.5% (36/122) and NDRV in 39.3% (48/122) of samples, with results fully validated by singleplex RT-PCR assays. Our study provides a reliable, specific, and reproducible tool for surveillance and epidemiological studies of MDRV and NDRV.

## 1. Introduction

Muscovy duck reovirus (MDRV) was first reported in South Africa in 1950, and the pathogen was first isolated and identified in 1972 [1,2]. Typical clinical manifestations in MDRV-infected ducks observed during necropsy include white pin-head necrotic foci in the liver and spleen, fibrinous pericarditis, and perihepatitis [3]. Since 1997, MDRV-induced disease has emerged as a major outbreak severely impacting the Muscovy duck industry in China, leading to high morbidity and mortality in ducklings aged 7–45 days [4]. Novel duck reovirus (NDRV), which appeared on the eastern coast of China in 2005, has since become endemic in duck farms [5]. NDRV infects various duck and goose species, causing necrosis in multiple immune organs, hemorrhage, diarrhea, and growth retardation [6,7]. Susceptibility is highest in ducks aged 3–25 days, with mortality rates ranging from 5% to 50% [8,9]. Notably, co-infections of MDRV and NDRV have been documented in clinical settings, complicating diagnosis and control efforts [10]. The spread of both viruses has resulted in substantial economic losses to the duck industry, underscoring the need for rapid and accurate diagnostic methods.

MDRV and NDRV are double-stranded RNA viruses with a double-capsid structure and no envelope, classified within the genus Orthoreovirus, family Reoviridae [3,11]. Their genomes consist of 10 segments, separable by SDS-PAGE into three large (L1–L3), three medium (M1–M3), and four small segments (S1–S4) [9,12]. The S3 gene encodes the sigma B protein, a major component of the viral capsid [13,14,15,16]. The nucleotide sequence identity of the S3 gene between NDRV and MDRV is just over 60%, while the deduced amino acid sequence identity is approximately 70% [9]. Due to this variability, the S3 gene has been widely used to differentiate between MDRV and NDRV [17,18].

Rapid and specific detection is essential for effective control of NDRV and MDRV infections. Although reverse-transcription loop-mediated isothermal amplification (RT-LAMP) and conventional RT-PCR are commonly used for detecting waterfowl reoviruses, these methods have limitations in throughput and specificity [19,20]. TaqMan-based real-time PCR assays have been developed for individual detection of MDRV or NDRV, offering high specificity, sensitivity, and reproducibility [21,22,23]. However, a method for simultaneous detection and differentiation of both viruses using duplex real-time RT-PCR has not been established. Therefore, this study aimed to develop a TaqMan-based duplex real-time quantitative RT-PCR assay for the concurrent detection and differentiation of MDRV and NDRV, and to evaluate its specificity, sensitivity, repeatability, and reproducibility.

## 2. Materials and Methods

### 2.1. Viral Strains

MDRV (CA strain) [16] and NDRV (JX20201210 strain) [24] were kept by Jiangsu Agri-Animal Husbandry Vocational College.

### 2.2. Reagents and Instruments

Nucleic acids were extracted with the TGuide Viral DNA/RNA Extraction Kit (TIANGEN Biotech, Beijing, China), using GenePure Pro 200 full automatic nucleic acid purification apparatus (Bioer Technology, Hangzhou, China). Primers and probes were diluted with TE buffer (Songon Biotech, Shanghai, China). RT-PCR was performed with One Step PrimeScript^TM^ RT-PCR Kit (Takara Bio, Beijing, China) in ABI 7500 Fast (Thermo Fisher Scientific, Waltham, MA, USA).

### 2.3. Primers and Probes

All available sequences of MDRV and NDRV were retrieved from GenBank for alignment via DNAMAN (see Supplementary Materials for retrieval criteria and sequence list in Table S1). Highly conserved regions from S3 gene of MDRV and NDRV were selected for primer and probe design (Primer Express 3.0 software). Sequences of primers and probes are listed in Table 1.

### 2.4. Standard Plasmid Construction

Recombinant plasmids pMD18-T-MDRV and pMD18-T-NDRV, each of which contains the target fragments of MDRV and NDRV, respectively, were synthesized as standard plasmids by General Biosystems Corp., Ltd. (Anhui, China). The ultraviolet absorbance of the standard recombinant plasmids at 260 nm and 280 nm was measured, and the plasmid copy number was calculated according to the following formula: plasmid concentration (copies/µL) = $\frac{\left(6.02\times 10^{23\;})\;\times \;(X\;ng/µL\;\times \;10^{-9}\right)}{plasmid\;length\;\left(bp\right)\;\times \;660}$. The standard plasmids were then serially diluted 10-fold from 10^5^ to 10^0^ copies/µL for optimizations of RT-PCR assays.

### 2.5. Optimization of the Singleplex RT-PCR Assay

The reaction mixture was prepared according to the One Step PrimeScript™ RT-PCR Kit protocol as follows: 10 µL of 2 × One-Step RT-PCR Buffer III, 0.4 µL of Ex Taq HS (5 U/µL), 0.4 µL of PrimeScript RT Enzyme Mix II, 0.8 µL of each primer (10 µM), 0.4 µL of probe (10 µM), 0.4 µL of ROX reference dye (50×), 2 µL of nucleic-acid template, and RNase-free water to a final volume of 5.6 µL, yielding a total reaction volume of 20 µL.

Thermal cycling conditions were set as follows: reverse transcription at 42 °C for 5 min, followed by an initial denaturation at 95 °C for 10 s (1 cycle). The PCR amplification comprised 40 cycles of 95 °C for 5 s and 60 °C for 30 s, with fluorescence data acquisition during the annealing step. To optimize annealing temperature, seven gradient points were evaluated: 50 °C, 52 °C, 55 °C, 58 °C, 60 °C, 62 °C, and 65 °C. For each temperature, fluorescence intensity and Ct values were compared to identify the most favorable conditions.

### 2.6. Optimization of the Duplex RT-PCR Assay

The duplex reaction system was optimized with the matrix method, by varying the volumes of primers (10 pmol/mL) and probes (10 pmol/mL). Primer volumes tested were 0.2, 0.4, 0.6, 0.8, 1.0 and 1.2 µL, while probe volumes evaluated were 0.2, 0.3, 0.4, 0.5, 0.6 µL.

### 2.7. Specificity of the Duplex RT-PCR Assay

To assess specificity of the assay, nucleic acids of positive samples for duck Tembusu virus (DTMUV), duck adenovirus 3 (DAdV-3), newcastle disease virus (NDV), duck plague virus (DPV), avian influenza viruse (AIV), duck circovirus (DuCV), duck plague virus (DPV), goose parvovirus (GPV), duck viral hepatitis virus type 1 (DHV-1) and duck viral hepatitis virus type 3 (DHV-3) were extracted and detected with the duplex RT-PCR assay. All the samples were previously stored in Jiangsu Agri-Animal Husbandry Vocational College.

### 2.8. Sensitivity of the Duplex RT-PCR Assay

To assess sensitivity of the assay, 10-fold serial dilutions (followed with further few 2-fold serial dilutions as needed) of a 1:1 mixture of standard plasmids containing the MDRV and NDRV targets were prepared and tested in triplicate. Limit of detection (LOD) as well as PCR amplification efficiency were determined based on the standard curves generated.

### 2.9. Repeatability of the Duplex RT-PCR Assay

To evaluate repeatability and reproducibility of the assay, both intra-assay and inter-assay were conducted. Plasmid mixture at three different concentrations (10^5^, 10^4^, and 10^3^ copies/µL) were prepared to serve as test samples. For the intra-assay, each concentration was tested in triplicate within a single run. For the inter-assay, triplicate tests of all three concentrations were performed across three independent experimental runs conducted on different days. The standard deviation (SD) and coefficient of variation (CV) were calculated to statistically evaluate the assay precision.

### 2.10. Clinical Sample Detection

A total of 122 tissue samples, collected from duck farms in Jiangsu Province, were utilized for detection. All samples were preserved and provided by Jiangsu Agri-animal Husbandry Vocational College. Each clinical sample was tested using both the newly developed duplex and singleplex RT-PCR assays. The results obtained from the two methods were compared to determine their concordance rate.

## 3. Results

### 3.1. Development and Optimization of the Duplex Real-Time RT-PCR Assay

The optimal annealing temperature for singleplex real-time RT-PCR was determined to be 60 °C. Following optimization with the matrix method, the final concentrations of primers and probes were established as 0.5 μmol/L and 0.3 μmol/L for MDRV, and 0.5 μmol/L and 0.2 μmol/L for NDRV, respectively (Table 2). The optimized amplification protocol comprised the following steps: reverse transcription at 42 °C for 5 min, followed by an initial denaturation at 95 °C for 10 s in one cycle; then a PCR amplification stage consisting of 40 cycles of denaturation at 95 °C for 5 s and annealing at 60 °C for 30 s, with fluorescence acquisition performed during the annealing phase of each cycle.

An experiment is deemed valid only if both negative and positive control reactions yield the expected outcomes. A negative result is assigned to samples that show no detectable fluorescence amplification curve throughout the amplification process. A positive result is defined by the presence of a fluorescence amplification curve with a Ct value ≤ 35. Samples displaying an amplification curve with a Ct value between 35 and 40 are considered indeterminate and must be re-extracted and re-tested for verification. If the repeat testing again produces a Ct value within the 35–40 range along with a visible amplification curve, the sample is conclusively interpreted as positive.

### 3.2. Specificity of the Duplex Real-Time RT-PCR Assay

**Figure 1:**
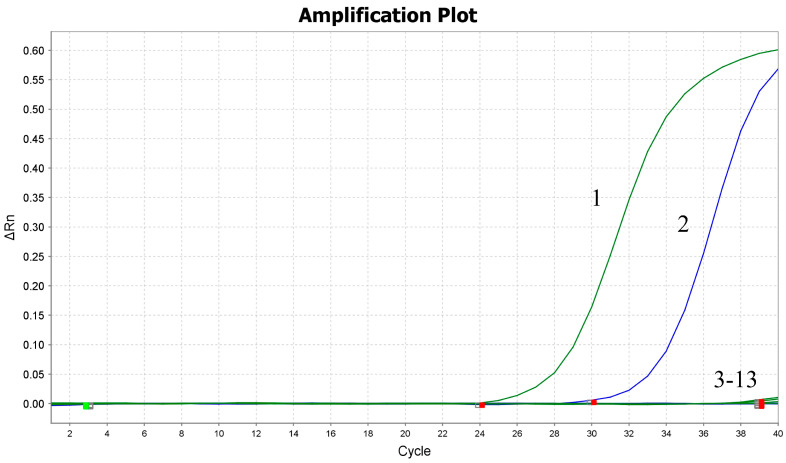
Specificity assessment of the duplex RT-PCR assay. The amplification plot depicts the cycle number on the *x*-axis and the ΔRn value on the *y*-axis. The amplification curves correspond to the following: curve 1, nucleic acid of NDRV; curve 2, nucleic acid of MDRV; curves 3–12, nucleic acids of DTMUV, DAdV-3, NDV, DPV, AIV, DuCV, DPV, GPV, DHV-1, and DHV-3; curve 13, negative control.

The specificity of the primers and probes was initially evaluated through in silico analysis employing the NCBI Primer-BLAST online tool (https://www.ncbi.nlm.nih.gov/tools/primer-blast/, accessed on 28 October 2025), which confirmed the uniqueness of the target sequences for each assay. Following the computational assessment, experimental specificity was further examined with nucleic acids of multiple viral strains, including MDRV, NDRV, DTMUV, DAdV-3, NDV, DPV, AIV, DuCV, DPV, GPV, DHV-1 and DHV-3. The assays specifically identified MDRV and NDRV, with no cross-reactivity observed with any non-target pathogens, thereby demonstrating high analytical specificity (Figure 1).

### 3.3. Sensitivity and Standard Curves of the Duplex Real-Time RT-PCR Assay

**Figure 2:**
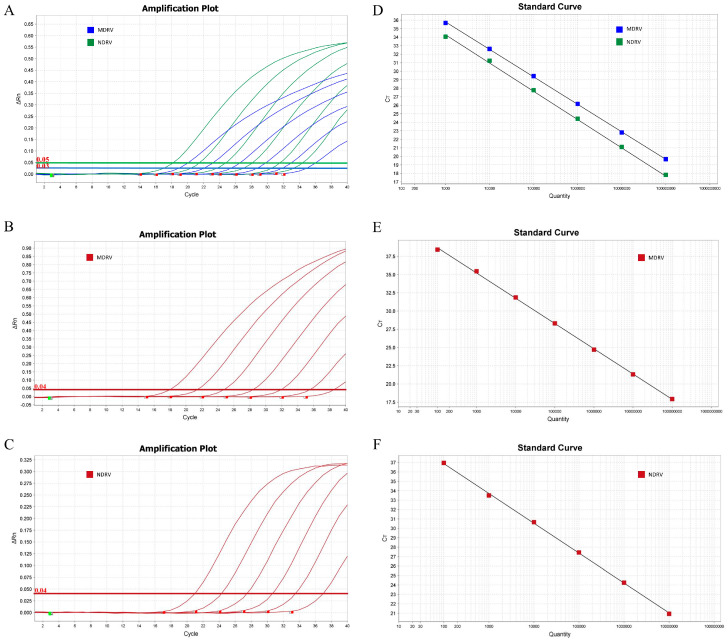
Amplification curves and standard curves of the duplex RT-PCR assay. The amplification curves for the duplex assay (**A**) as well as singleplex assays (**B**,**C**) were generated using recombinant standard plasmids pMD18-T-MDRV and pMD18-T-NDRV. The standard curves (**D**–**F**) were generated from the amplification curves. In (**A**), for the curves from left to right, the plasmid concentrations ranged from 10^7^ to 10^2^ copies/μL for each target; In (**B**,**C**), for the curves from left to right, the plasmid concentrations ranged from 10^7^ to 10^1^ copies/μL.

To evaluate the sensitivity of the assay, 10-fold serial dilutions of a 1:1 mixture of standard plasmids containing the MDRV and NDRV targets (ranging from 10^7^ to 10^2^ copies/μL per target) were tested in triplicate. Further few 2-fold serial dilutions were tested as well, to carefully assess the limit of detection. Standard curves for both the duplex and singleplex assays were generated by plotting the Ct values against the logarithm of the initial template copy number (Figure 2). The PCR amplification efficiencies ranged from 94% to 107%, and all correlation coefficients (R^2^) were ≥0.999. Specifically, for the MDRV assays, efficiencies were 94.49% (singleplex) and 104.04% (duplex), with R^2^ values of 1 and 0.999, respectively. For the NDRV assays, efficiencies were 106.67% (singleplex) and 100.11% (duplex), both with R^2^ values of 0.999. These results demonstrate that the multiplex format does not inhibit PCR sensitivity compared to the singleplex assays. The limit of detection for both targets was determined to be less than 30 copies (Table 3).

### 3.4. Repeatability and Reproducibility of the Duplex Real-Time RT-PCR Assay

The intra- and inter-assay variability of Ct values were thoroughly evaluated. As summarized in Table 4, the coefficients of variation (CV) for both MDRV and NDRV were consistently below 1.5%, indicating that the developed duplex real-time RT-PCR assay exhibits excellent repeatability and high stability.

### 3.5. Application and Detection of the Duplex Real-Time RT-PCR Assay

A total of 122 clinical duck tissue samples were analyzed using the developed duplex real-time RT-PCR assay. The assay detected MDRV and NDRV in 29.51% (36/122) and 39.34% (48/122) of the samples, respectively, and no co-infections were observed. To validate these findings, all samples were re-tested with the singleplex real-time RT-PCR assays, and the results were in complete agreement (Table 5).

## 4. Discussion

In this study, we developed and validated a TaqMan-based duplex real-time RT-PCR assay for the simultaneous detection and differentiation of MDRV and NDRV. The performance of our assay was rigorously evaluated, and its clinical utility was demonstrated through the testing of field samples. A critical assessment of our method against existing technologies and a detailed interpretation of our findings are presented below.

A key strength of our duplex assay lies in its competitive sensitivity and specificity when compared to other molecular diagnostics. The limit of detection (LOD) of our assay (13–25 copies/µL) is comparable to, and in some cases superior to, previously reported qPCR assays for MDRV or NDRV, which reported LODs ranging from 10 to 100 copies/µL [21,22,23,25,26]. While recent advancements have introduced highly sensitive techniques like digital PCR (dPCR), which offers absolute quantification and can detect single copies, our TaqMan qPCR provides a more accessible, cost-effective, and high-throughput solution for routine diagnostic and surveillance purposes [10,27]. The primary trade-off for multiplexing was a slight reduction in sensitivity for MDRV in the duplex format (LOD of 25 copies) compared with its single-plex counterpart (LOD of 10 copies). This loss of less than one log is acceptable for a diagnostic context, as the duplex LOD remains well within a clinically relevant range and is offset by the efficiency and cost-effectiveness of testing two pathogens in a single reaction.

The assay demonstrated excellent robustness, with amplification efficiencies between 100.11% and 106.67% and R^2^ values > 0.999, indicating highly reliable quantification over a wide dynamic range. The minimal intra- and inter-assay variability (CV < 1.5%) underscores its suitability for reproducible application across different runs and operators. Notably, the amplification efficiency for MDRV improved in the duplex format (104.04%) compared with the single-plex (94.49%), likely due to optimized primer–probe interactions in the combined reaction environment. This consistency and reliability are paramount for a clinical setting, where the assay can reliably categorize samples as positive (Ct ≤ 35) or requiring retesting (Ct 35–40), ensuring both high throughput and diagnostic accuracy.

Application to 122 clinical samples revealed a higher detection rate for NDRV (39.34%) than for MDRV (29.51%), with no co-infections observed. This epidemiological pattern aligns with previous observations in duck populations where NDRV predominates over MDRV in certain regions [23,26,28]. A critical validation step was the 100% concordance between the duplex and single-plex assays for all clinical samples; no samples changed categorization between the two methods, and Ct values for positive samples were highly comparable, with minimal differences. These results confirm that the duplex assay does not compromise diagnostic certainty and is a robust tool for field surveillance.

In conclusion, this study successfully achieved its aim of establishing a reliable, specific, and reproducible duplex real-time RT-PCR for the simultaneous detection and differentiation of MDRV and NDRV. While acknowledging the existence of alternative, highly sensitive methods such as dPCR, our assay presents an optimal balance of performance, practicality, and cost-efficiency. It provides a valuable tool for large-scale epidemiological studies and routine diagnostic monitoring, ultimately contributing to the improved control of these economically significant pathogens in the duck industry.

## Figures and Tables

**Table 1:** Sequences of primers and probes.

Virus	Primer/Probe	Sequence (5′–3′)	Size (bp)	Reference Strain Genbank No.
MDRV	MDRV-F	TCCAGTACTTTCAGGCACCTCAT	111	DQ643971
	MDRV-R	TGAAACCGCAGGTTCAGGAT		
	MDRV-P	VIC-TGGCGCATCCATCGCCTCG-BHQ1		
NDRV	NDRV-F	GCTAGATGTGAATCGCATAACGA	87	GQ888710
	NDRV-R	GCCATAAAGGAAGCAGAAGCA		
	NDRV-P	NED-TGACGTTGCTATGGTAACTCCTTCTGCTGC-BHQ2		

**Table 2:** Optimal reaction system for the duplex RT-PCR assay.

Reagents	Volume (µL)	Final Concentration (µM)
2 × One-Step RT-PCR buffer III	10	
MDRV primer set (10 µM)	1	0.5
NDRV primer set (10 µM)	1	0.5
MDRV probe (10 µM)	0.6	0.3
NDRV probe (10 µM)	0.4	0.2
ROX (50×)	0.4	
Ex Taq HS (5 U/µL)	0.4	
PrimeScript RT Enzyme Mix II	0.4	
Nucleic acid template	2	
RNase free water	Up to 20	

**Table 3:** Parameters of the standard curves.

Virus	Amplification Efficiency (%)		R^2^		Limit of Detection (LOD) (Copies)	
	Singleplex	Duplex	Singleplex	Duplex	Singleplex	Duplex
MDRV	94.49	104.04	1	0.999	10	25
NDRV	106.67	100.11	0.999	0.999	10	13

**Table 4:** Repeatability and reproducibility of the duplex RT-PCR assay.

Virus	Concentration (Copies/µL)	Intra-Assay			Inter-Assay		
		Ct (Mean)	SD	CV (%)	Ct (Mean)	SD	CV (%)
MDRV	10^5^	26.58	0.35	1.33	26.41	0.20	0.75
	10^4^	29.14	0.06	0.21	29.28	0.11	0.36
	10^3^	32.76	0.09	0.26	32.38	0.28	0.86
NDRV	10^5^	24.72	0.14	0.57	24.49	0.05	0.22
	10^4^	27.39	0.37	1.36	27.59	0.33	1.19
	10^3^	31.45	0.06	0.18	31.26	0.12	0.39

**Table 5:** Comparison of Ct values from singleplex and duplex assays.

Virus	Assay Format	No. of Positive Samples	Ct Value Range	Mean Ct ± SD	Concordance Between Assays
MDRV	Singleplex	36	22.4–34.1	27.8 ± 3.2	100%
	Duplex	36	22.7–34.5	28.1 ± 3.3	
NDRV	Singleplex	48	20.8–33.6	25.9 ± 3.5	100%
	Duplex	48	21.1–33.9	26.2 ± 3.6	

## Data Availability

All the Supporting Information has been provided in the text. Any other supporting files, if requested, can be provided by the corresponding authors.

## Supplementary Materials

The following supporting information can be downloaded at: https://www.mdpi.com/article/10.3390/pathogens14121231/s1, Table S1: Representative List of MDRV and NDRV S3 Gene Sequences Retrieved from GenBank for Primer and Probe Design.

## Abbreviations

The following abbreviations are used in this manuscript:

MDRV	Muscovy duck reovirus
NDRV	Novel duck reovirus
RT-LAMP	Reverse-Transcription Loop-mediated Isothermal Amplification
RT-PCR	Reverse-Transcription Polymerase Chain Reaction
SDS-PAGE	Sodium Dodecyl Sulfate-Polyacrylamide Gel Electrophoresis
LOD	Limit of detection
DTMUV	Duck Tembusu virus
DAdV-3	Duck adenovirus 3
NDV	Newcastle disease virus
DPV	Duck plague virus
AIV	Avian influenza viruse
DuCV	Duck circovirus
GPV	Goose parvovirus
DHV-1	Duck viral hepatitis virus type 1
DHV-3	Duck viral hepatitis virus type 3
Ct	Cycle threshold
CV	Coefficients of variation
SD	Standard Deviation
