# Supplementary material for: Development of a TaqMan-Based Duplex Real-Time Quantitative RT-PCR for Detection and Differentiation of Muscovy Duck Reovirus and Novel Duck Reovirus
Source: Pathogens. 2025 Dec 2;14(12):1231. doi: 10.3390/pathogens14121231 (PMC12735753; doi:10.3390/pathogens14121231)
Supplement: Supplementary file 1 [file pathogens-14-01231-s001.zip › pathogens-3983026-supplementary.pdf]

Details of Sequence Retrieval and Analysis for Primer and Probe Design

The design of the specific primers and TaqMan probes targeting the S3 gene of Muscovy duck reovirus (MDRV) and novel duck reovirus (NDRV) was based on a comprehensive analysis of all available sequences in the public database.

1. **Database and Retrieval Date:** All related viral sequences were retrieved from the **NCBI GenBank** database on **March 1, 2025**.
2. **Retrieval Strategy and Criteria:** The retrieval was performed using the **NCBI Nucleotide BLAST** tool and the **NCBI Virus** portal. The search was conducted using the following keywords and criteria:
   1) **Keywords:** "Muscovy duck reovirus S3", "Novel duck reovirus S3", "Duck orthoreovirus", "sigma B protein".
   2) **BLAST Search:** Additionally, a BLASTn search was performed using several reference S3 gene sequences (e.g., GenBank Accession No. DQ643971 for MDRV and GQ888710 for NDRV) to identify homologous sequences with high percentage identity (>80%).
   3) **Inclusion Criteria:** Only complete or near-complete coding sequences of the S3 gene from identified MDRV and NDRV strains were selected for alignment.
3. **List of Retrieved Sequences:** A total of 84 sequences (17 for MDRV and 67 for NDRV) were retrieved and compiled for multiple sequence alignment. A representative list of these sequences, including the GenBank Accession Number and Virus Strain, is provided in the table below. The complete list is available upon request.

Table S1 Representative List of MDRV and NDRV S3 Gene Sequences Retrieved from GenBank for Primer and Probe Design.

Virus	GenBank Accession No.	Virus Strain	Country	Year
MDRV	DQ643971	S12	China	2007
MDRV	JX478268	J18	China	2012
MDRV	OK626891	HN21	China	2021
NDRV	GQ888710	NP03	China	2009
NDRV	MH510263	DH13	China	2019
NDRV	KJ879932	SD12	China	2020

4. **Sequence Analysis:** The retrieved sequences were aligned using the DNAMAN software (version 8.0, Lynnon Biosoft). The alignment was visually inspected to identify highly conserved regions unique to either MDRV or NDRV, which were then used as targets for the design of virus-specific primers and TaqMan probes (as listed in Table 1 of the main text). The specificity of the designed oligonucleotides was further verified *in silico* using the NCBI Primer-BLAST tool to ensure no significant homology with non-target organisms.
